# Validity of the Isometric Contraction Test of the Masticatory Muscles for Diagnosis of Muscular Temporomandibular Disorders

**DOI:** 10.3390/diagnostics12081861

**Published:** 2022-08-01

**Authors:** Marcos Iglesias-Peón, Juan Mesa-Jiménez, César Fernández-de-las-Peñas, Jorge Rojas-García, Daiana Priscila Rodrigues-de-Souza, Francisco Alburquerque-Sendín

**Affiliations:** 1 Osteopatía y Fisioterapia Guadalajara, 19005 Guadalajara, Spain; z12igpem@uco.es (M.I.-P.); clinicaofgu@hotmail.com (J.R.-G.); 2 Doctoral Program in Biomedicine, University of Córdoba, 14004 Córdoba, Spain; 3 Department of Physical Therapy, Universidad San Pablo CEU, Boadilla del Monte, 28668 Madrid, Spain; jmesaj@ceu.es; 4 Department of Physical Therapy, Occupational Therapy, Rehabilitation and Physical Medicine, Universidad Rey Juan Carlos, Alcorcón, 28922 Madrid, Spain; cesar.fernandez@urjc.es; 5 Department of Nursing, Pharmacology and Physical Therapy, Faculty of Medicine and Nursing, University of Córdoba, 14004 Córdoba, Spain; falburquerque@uco.es; 6 Maimonides Biomedical Research Institute of Cordoba (IMIBIC), 14004 Córdoba, Spain

**Keywords:** temporomandibular disorders, test accuracy, muscle dysfunction, orofacial pain

## Abstract

In recent years, the Diagnostic Criteria for Temporomandibular Pain Disorders (DC/TMD) has been questioned, mainly because of the dependence on the muscular pressure needed to be applied during the clinical examination. Therefore, it is necessary to establish improvements in diagnostic strategies for DC/TMD of axis I. The aim of this study was to determine the validity of the Isometric Contraction Test of the masticatory muscles (ICTest) to diagnose DC/TMD of axis I. Forty (n = 40) patients with muscular TMD (myalgia in any of its subtypes), as well as forty age and sex matched controls, participated. They were diagnosed according to DC/TMD of axis I and performed the ICTest in a single session. Sensitivity, specificity, positive (PPV) and negative predictive values (NPV), and positive (LR+) and negative likelihood ratios (LR−) of the ICTest compared with the DC/TMD of axis I were calculated. The ICTest showed a specificity of 95% for the diagnosis of myalgia, and between 94.9% and 96.8% for all subtypes in relation to the DC/TMD of axis I. For sensitivity, lower values were obtained, that is, 90.0% for myalgia, and losing sensitivity depending on the type of myalgia. The LR+ was over 10 for all diagnoses, with the exception of myofascial pain with referral, which was lower. When addressing the LR−, the myofascial diagnosis was the only one below 0.2. According to the results, the ICTest could be considered a valid procedure to diagnose subjects with muscular TMD in a clinical setting.

## 1. Introduction

Temporomandibular pain is considered the most common cause of pain of non-dental origin [1]. The American National Institute of dental to craniofacial research (NIDCR), in its last revision of 2018, determined a prevalence between 5% and 12% for pain at the temporomandibular joint (including muscular disorders) [2]. In addition, the highest percentage of individuals affected is young people, something unusual in chronic pain. Finally, it is estimated that expenses related to temporomandibular pain in the United States of America have reached 4 billion dollars in the last decade [3]. A significant amount of these efforts is focused on determining effective therapeutic approaches, as recently reported [4,5], including treatments for TMD of muscular origin [6,7].

Schifmann et al. [3] added new diagnoses to axis I [8], which included muscle alterations originated in the TMD, with this axis being the most prevalent in the population, with 45.3%. Axis I taxonomy divides muscle pain into four major subclasses: myalgia, tendinosis, myositis, and muscle spasm. Within the myalgia subclass, a new classification provides a further subdivision, with three different subtypes: local myalgia, myofascial pain, and myofascial pain with referral [3]. The diagnostic Criteria for Temporomandibular Disorders (DC/TMD) of axis I have shown high validity, specifically for pain of muscular origin and for the different subtypes of myalgia [3]. The diagnosis of myalgia has a sensitivity of 0.9 (95% CI 0.87–0.94) and a specificity of 0.99 (95% CI 0.97–1.00). Myofascial pain with referral has a sensitivity of 0.86 (95% CI 0.79–0.94) and a specificity of 0.98 (95% CI = 0.97–0.99). However, the sensitivity and specificity of the two other subtypes (local myalgia and myofascial pain) have not been established in DC/TMD. Furthermore, the DC/TMD evaluation protocol requires a priori training of the assessors to acquire the necessary skills for its clinical application. 

In recent years, the DC/TMD has been questioned, given the high dependence on the muscular pressure needed to be applied during the clinical examination [9]. Even the palpation of certain muscles is questioned because of the difficulty in accesses them (owing to their anatomical location) or the characteristics of the palpated tissue that can cause pain [10]. In fact, studies have shown that, for clinical purposes, both dynamic and static tests are more powerful than palpatory tests [11].

A rigorously developed assessment of the instruments used in physical therapy continues to be a challenge [12]. In relation to TMD, the relevance of determining the metric properties of each new evaluation tool, in terms of validity and reliability, is high in clinical settings and research [13,14]. In fact, it has been reported that a better understanding and application of DC/TMD could be needed [15]. Therefore, it is necessary to establish improvements in diagnostic strategies for DC/TMD, and especially in the diagnosis of axis I. In this regard, different temporomandibular pain evaluation tools have emerged with clinical practice purposes. Previous research has shown that various clinical tests (active, passive, joint sounds, isometrics, joint mobility, joint provocation, and so on) obtain excellent reliability data in patients with TMD, given that 4–5 tests were positive out of the 7 tests conducted. Further, the general isometric contraction tests determined a high inter-examiner reliability with a kappa index (95% CI 0.50–0.92) [16]. Osiewicz et al. compared the values of the dynamic and static tests to the palpatory tests in patients with TMD, showing high reliability values (intra-examiner and inter-examiner evaluation ICC, ranging from 0.63 to 0.70 for mouth opening, 0.68–0.73 for mouth closing, and 0.52–0.72 for protrusion, respectively [11]). However, the validity of these tests to diagnose TMD had to be established.

Thus, the aim of this study was to determine the validity of the Isometric Contraction Test of the masticatory muscles (ICTest) to diagnose myalgia TMD and its subtypes, that is, local myalgia, myofascial pain, and myofascial pain with referral, according to DC/TMD of axis I.

## 2. Methods

### 2.1. Design

We conducted a validation study of a diagnostic test, with non-probabilistic recruitment of consecutive cases. The Research Ethics Committee of Córdoba approved this project (protocol code 5372-2022). All participants signed the informed consent form before their inclusion in the study.

### 2.2. Subjects

Eighty subjects participated in the study (36 men and 44 women), in an age range of 20 to 67 years old, recruited at the Biosanitary campus of the University of Córdoba (Spain) and a private physiotherapy clinic in Guadalajara city (Spain) in three different ways: (i) subjects who requested treatment and manifested temporomandibular pain, (ii) patients with habitual temporomandibular pain contacted by telephone, or (iii) by social networks. The inclusion criteria were as follows: 18 years or more; pain in the masticatory muscles diagnosed as DC/TMD of the axis I [3], for the subjects of the case group; or no pain history in the masticatory muscles or any other orofacial area, for the subjects of the control group. The exclusion criteria were common for both groups, as follows: suffering from acute temporomandibular joint block; history of TMD surgery; not understanding basic commands necessary for the application of the test; previous medical diagnosis of headache; spinal pain in the last three months; or history of autoimmune diseases (rheumatoid arthritis, lupus, psoriasis). Subjects of the control group were matched to the cases by sex and age (±5 years).

### 2.3. Procedures

All subjects received a complete explanation of the objectives of the study and an informed consent was provided and signed. Afterwards, the subjects were submitted to the DC/TMD “CS3 Symptom Questionnaire”, in its validated Spanish version [17], as the gold standard procedure, to be diagnosed as cases or controls and, accordingly, to be included in one of the study groups.

**Figure 1:**
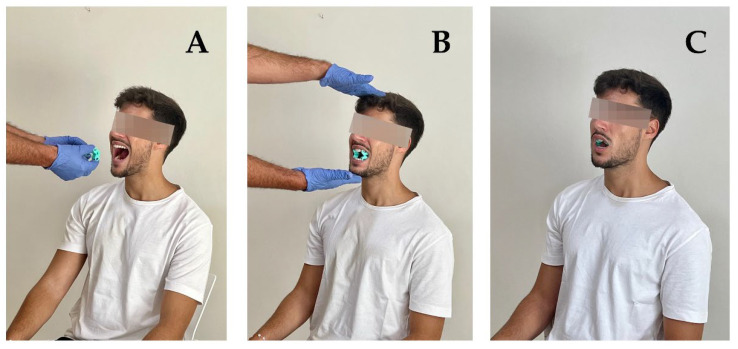
Isometric contraction test of the occlusive masticatory muscles. (**A**) First, the subject opens the mouth; (**B**) second, the assessor inserts the teethers in the subject’s mouth; (**C**) third, the subject clenches the teeth during 40 s.

Subsequently, the ICTest was applied following a standard protocol for all subjects. Each subject was asked to sit in a chair and received the following instructions: “From this sitting position, I am going to insert these two teethers between the lower molar and premolar teeth on both sides of your mouth. Later, with the teethers on the teeth, I am going to ask you to try to clench the teeth as much as you can for 40 s. I will tell you when the time is up. If during the test you feel any pain, you should indicate the area of the pain with your finger. When the time is up, I will remove the teethers and I will ask you if the pain is at a specific point, in an area, or if it describes a certain path. If the pain is unbearable at any moment, you can stop the contraction whenever you want. After the test, and only if there was pain when doing it, you must say if the pain experienced during the test reproduced any symptom that you have experienced in the last 30 days” (Figure 1).

**Figure 2:**
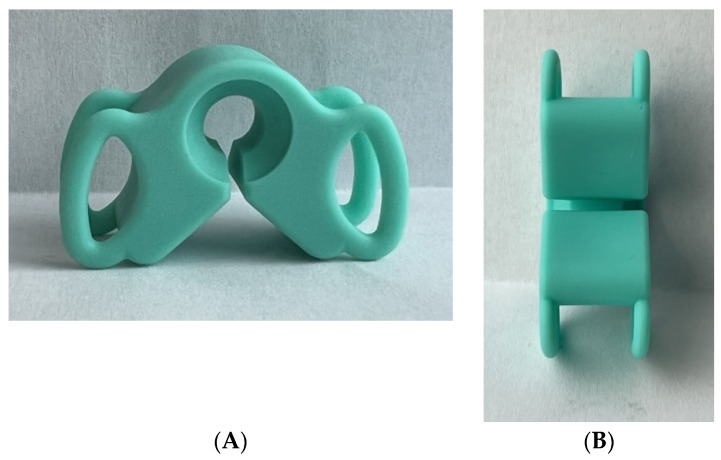
Teethers (Morde block teethers size S (Bader laboratory, reference 11/022)) used in the ICTest. (**A**) Lateral view; (**B**) anterior view.

The location of the two teethers (Morde block teethers size S model (Bader laboratory, reference 11/022), dimensions 3.3 × 2.8 × 1.8 cm) (Figure 2) was symmetrical, from the last molar to the premolar teeth on each side. Two teethers were used in order to achieve the most dental occlusion possible. This test was considered positive when pain appeared, and the elicited pain during the test was similar to or like any symptom the patient experienced in the last month. The test was considered negative when no pain appeared or when the elicited pain during the test was not similar to or like any symptom the patient experienced in the last month [9].

Each type of myalgia (local myalgia, myofascial pain, and myofascial pain with referral) was identified independently, according to DC/TMD. Two physical therapists, with more than 5 years of clinical experience in TMD, were trained for the study. Ten subjects, five with DC/TMD of axis I and five without DC/TMD of axis I, who were not finally included in the main analysis, participated in the training. A digital algometer was used to establish the manual pressure to be exerted on each pressure point (1 Kg/cm^2^ in external exploration muscles and 0.5 Kg/cm^2^ in intraoral exploration muscles) for DC/TMD application. One of the therapists, trained in orofacial and temporomandibular pain and in the exploration of DC/TMD, collected the data corresponding to this gold standard [3]. The second therapist, who was blinded to the DC/TMD findings of each subject, applied the ICTest, as previously described.

### 2.4. Sample Size Calculation

The sample size was estimated considering the comparison of two proportions (Chi-squared statistic with continuity correction) [18] in order to detect a sensitivity of 0.8 and a 1-specificity of 0.2, with a 1:1 proportion between cases and controls, and a two-tailed test with an alpha level of 0.05 and a power of 90% (Tamaño de la muestra 1.1^®^). Thirty-two subjects were initially required. To assume imbalances when specific myalgia types were analyzed, 40 subjects were included in each group. 

### 2.5. Statistical Analysis

Data were analyzed with the statistical package IBM-SPSS (version 25.0; SPSS, Chicago, IL, USA). The results are expressed as the mean, standard deviation, and 95% confidence interval for quantitative data, and frequencies and percentages for qualitative data. The Kolmogorov–Smirnov test was used to verify the normality of the demographic characteristics data of the subjects in both groups.

To analyze the differences in the sociodemographic data of both groups, unpaired two-tailed Student’s *t*-tests were used for parametric variables and the Mann–Whitney U test was used for non-parametric variables.

In addition, the sensitivity, specificity, positive (PPV) and negative predictive values (NPV) expressed as percentage, and positive (LR+) and negative likelihood ratios (LR−) of the ICTest compared with the DC/TMD as the gold standard of axis I were obtained [19,20]. Good performance for a diagnostic test is considered when the sensitivity and specificity were >0.8 [21], and when LR+ > 10 and LR− < 0.1 [22]. Youden’s index was also reported [23], and was considered acceptable when >0.5 [24].

All statistical tests were calculated with a 95% confidence interval. The values were considered significant if *p*-value < 0.05.

## 3. Results

A total of 40 individuals with masticatory muscles pain, according to DC/TMD of axis I (18 men, 22 women; age: 45.9 ± 13.8 years old; BMI: 24.4 kg/m^2^) and age of 40 and sex-matched controls (18 men, 22 women; age: 48.5 ± 11.5 years old; BMI: 23.6 kg/m^2^) were included. Patients showed a mean history of symptoms of 8.6 years.

The validity values for the ICTest with respect to the DC/TMD of axis I showed a specificity of 95% for myalgia (Table 1), and between 94.9% and 96.8% for all subtypes of myalgia (Table 2). The same trend was identified for NPV, with values ranging between 82.4% and 95.5% for all diagnoses. The sensitivity values were slightly lower, that is, 90% for myalgia, and 81% and 78.6% for local myalgia and myofascial pain, respectively. The PPV showed a similar trend, ranging from 94.7% for myalgia in general to 66.7% for myofascial pain with referral (Table 1 and Table 2).

The LR+ was over 10 for all diagnoses, with the exception of the myofascial pain with referral, which achieved 7.41. For the LR-, the overall myofascial diagnosis was the only one with data below 0.2, while local myalgia and myofascial pain ranged between 0.2 and 0.5. In fact, myofascial pain with referral was the only diagnosis that showed a Youden index below 0.6 (Table 1 and Table 2).

## 4. Discussion

The ICTest is a valid tool to identify subjects according to DC/TMD of axis I. The specificity of the test is high for overall diagnosis of myalgia as well as for each subtype, that is, for local myalgia, myofascial pain, and myofascial pain with referral. Lower values were identified for sensitivity, with myofascial pain with referral below 50%. The ICTest has been found to be applicable in clinical practice owing to its innocuousness, high speed, and low cost, as no patient reported discomfort and there was no dropout, which are desirable characteristics for any evaluation tool [25]. In the same way, the ICTest does not depend on the examiner’s capacity to apply muscular resistance or pressure in a standardized fashion, with the patients themselves exerting pressure on the teether. Furthermore, the ICTest includes pain provoked by masticatory structures, which is a desirable criterion to apply in the confirmation of TMD [9,10].

The ICTest was more specific than sensitive, meaning that its use could be more interesting to confirm a diagnosis than to exclude it in a screening process. In fact, a high proportion of false negatives was detected when the subtypes of myalgia were considered, while the false positive ratio remained stable along the different diagnoses, or even decreased, as was the case in myofascial pain with referral. Besides, the positive and negative likelihood ratios demonstrated that a positive ICTest is more capable of ratifying the TMD into the axis I than a negative ICTest is of rejecting the diagnosis, confirming the asymmetry of what a clinician must think when a positive ICTest is found (the probability of the subject suffering DC/TMD of axis I is large) or when a negative ICTest is found (the probability of the subject to not suffering DC/TMD of axis I is moderate or even small) [19]. Finally, the balance between sensitivity and specificity determined that both the overall myalgia and local myalgia and myofascial pain subtypes have an acceptable diagnosis ability in clinical settings (Youden’s index >0.5), while this approach is unacceptable for myofascial pain with referral [24]. These data do not differ from those of other validity studies in physiotherapy, where the specificity is higher than the sensitivity, as reported for the diagnosis of myofascial pain in upper quarter musculature [26], and the own DC/TMD for myalgia and myofascial pain with referral [3]. Nevertheless, not all of diagnostic tests have these characteristics, as it is possible to find studies that obtain values where the sensitivity is greater than the specificity, as in certain studies of cervical spine hypomobility [27]. In summary, it could be proposed that symptoms derived from masticatory muscles are able to confirm a TMD, but their absence does not exclude TMDs, which is relevant in any given clinical setting. Maybe a more complex approach is necessary, as recently stated in an international consensus determining that evaluations through mandibular movements, muscle palpation, and cervical assessment were the preferred tests for a correct complete evaluation of the patient, as they achieved an appropriate cataloging of the different TMD subgroups, and in this way, it was possible to collaborate with other disciplines using the DC/TMD [28].

Accordingly, the results indicate that this test is valid compared with the gold standard (DC/TMD), mainly for the diagnosis of myalgia and local myalgia, and not so much for myofascial pain or myofascial pain with referral. This could be because of the fact that referred muscle pain is more complex in origin and in its characteristics. The mechanisms underlying myofascial syndromes and myofascial trigger points are not completely described [29,30], although there are current theories that relate peripheral nociception to central sensitization, also involving the microvascular system and neurotransmitters at a the cellular level [31], and even age as a relevant factor that determines their origin [32]. Furthermore, there are theories that include the role of the fascia in myofascial pain and referred pain [33]. It has also been suggested that an additional research consideration could be whether pain spreading and referral are truly reflections of central factors, which would justify a need to separate these conditions from peripheral local myalgia to make the classification of subgroups more homogenous [10]. Perhaps, this component, which implies the individualization of pain related to diverse personal aspects, could be the cause of the greater heterogeneity of triggering pain when a muscle contraction is requested. In other words, it seems to indicate that local pain is more easily detectable by muscle contraction than myofascial pain or myofascial pain with referral, as has also been reported for the identification and classification of myofascial trigger points by manual palpation in shoulder muscles, mainly in the symptomatic side of subacromial impact syndrome [34]. The current results also confirm those obtained in DC/TMD, whereas the sensitivity and specificity values for myofascial with referral pain were lower than those for myalgia, as previously stated, where the values for local myalgia and myofascial pain were not established [3]. The influence of the heterogeneity in palpation techniques and its description [9,29] could be other source of bias in DC/TMD of axis I that could have influenced our results. Although the convenience of palpation in DC/TMD diagnosis exceeds the aim of this study, it should be recognized that normal temporomandibular joint function implies, among other features, pain-free muscle contraction [25], which questions the palpation as the only source of muscle symptoms in TMD assessment.

We should recognize some limitations of the study. First, sex has been identified as a factor associated with orofacial pain, but there was no gender consideration in this study. Second, although the performance of ICTest is probably not very assessor-dependent, the interpretation of the classification of the myofascial subcategories could be questioned [9]. Moreover, no reliability analysis was performed, which is useful for the evaluation of the orofacial area [13]. Third, different muscles that could promote TMD pain, such as masseter, temporalis, and internal pterygoid, were not taken into account, which could afford new and relevant data. Fourth, although the study population characteristics influence the results of validity studies [9], other specific sources of pain that could be related to TMD, such as head and neck pain [35,36] or fibromyalgia [29], were not even considered. Fifth, both the ICTest and DC/TMD of axis I are dichotomous tests, which does not allow them to establish any cut-off points or quantitative results. Finally, the psychological condition of a patient, in terms of stress, exercise habits, or comorbid medical conditions, could influence the patient’s sensitization to pain when performing the test [37,38]. Further investigations into etiology and sociocultural factors, and their potential contribution to taxonomy and diagnosis, are required, particularly within the context of a complex disease such as TMD [15].

## 5. Conclusions

The ICTest is a valid tool to diagnose subjects with DC/TMD of axis I. The ability of the test to confirm the presence of overall myalgia was higher than the ability to detect possible cases or subtypes. When the type of myalgia was considered, local myalgia was better diagnosed than myofascial pain or myofascial pain with referral, which are difficult to address (low values of sensitivity and negative LR). This study shows that the ICTest could be applicable in clinical practice thanks to its innocuousness, high speed, and low cost, although further knowledge about its reliability is required.

## Figures and Tables

**Table 1:** Validity results for the ICTest with respect to DC/TMD of axis I (myalgia).

	**DC/TMD Axis I +**	**DC/TMD Axis I −**	**Total**		**LR+**	**LR−**
**ICTest +**	36	2	38	PPV = 94.7 (82.7–98.5)	18.00 (4.64–69.77)	0.11 (0.04–0.27)
**ICTest −**	4	38	42	NPV = 90.5 (77.9–96.2)		
**Total**	40	40				
	Sensitivity = 90.0 (76.9–96.0)	Specificity = 95.0 (83.5–98.6)		Youden index = 0.9		

PPV: positive predictive value; NPV: negative predictive value; LR+: positive likelihood ratio; LR−: negative likelihood ratio; sensitivity, specificity, VP+, VP−, LR+, LR− expressed as value (95% confidence interval).

**Table 2:** Validity results for the ICTest with respect to the types of myalgia according to DC/TMD.

**LOCAL MYALGIA**										
	DC/TMD Axis I +	DC/TMD Axis I −	Total	Sensitivity	Specificity	PPV	NPV	LR+	LR−	Youden index
**ICTest +**	17	3	20	81.0 (60.0−92.3)	94.9 (86.1−98.3)	85.0 (64.0−94.8)	93.3 (84.1−97.4)	15.92 (5.19−48.88)	0.20 (0.08−0.49)	0.8
**ICTest −**	4	56	60							
**Total**	21	59								
**MYOFASCIAL PAIN**										
	DC/TMD Axis I +	DC/TMD Axis I −	Total	Sensitivity	Specificity	PPV	NPV	LR+	LR-	Youden index
**ICTest +**	11	3	14	78.6 (52.4−92.4)	95.5 (87.5−98.4)	78.6 (52.4−92.4)	95.5 (87.5−98.4)	17.29 (5.53−53.99)	0.22 (0.08–0.62)	0.7
**ICTest −**	3	63	66							
**Total**	14	66								
**MYOFASCIAL PAIN WITH REFERRAL**										
	DC/TMD Axis I +	DC/TMD Axis I −	Total	Sensitivity	Specificity	PPV	NPV	LR+	LR-	Youden index
**ICTest +**	4	2	6	23.5 (9.6−47.3)	96.8 (89.1−99.1)	66.7 (30.0−90.3)	82.4 (72.2−89.4)	7.41 (1.48−37.10)	0.79 (0.58−1.07)	0.2
**ICTest −**	13	61	74							
**Total**	17	63								

PPV: positive predictive value; NPV: negative predictive value; LR+: positive likelihood ratio; LR−: negative likelihood ratio; sensitivity, specificity, VP+, VP−, LR+, LR− expressed as value (95% confidence interval).

## Data Availability

The data presented in this study are available upon reasonable request from the corresponding author.
